# Comparison of parasite sequestration in uncomplicated and severe childhood *Plasmodium falciparum* malaria^☆^

**DOI:** 10.1016/j.jinf.2013.04.013

**Published:** 2013-09

**Authors:** Aubrey J. Cunnington, Michael T. Bretscher, Sarah I. Nogaro, Eleanor M. Riley, Michael Walther

**Affiliations:** a Department of Immunology and Infection, Faculty of Infectious and Tropical Diseases, London School of Hygiene and Tropical Medicine, Keppel Street, London WC1E 7HT, UK; b Medical Research Council (UK) Laboratories, PO Box 273, Banjul, Gambia; c Section of Paediatrics, Department of Medicine, Imperial College, Norfolk Place, London W2 1PG, UK; d Immune Regulation Section, Laboratory of Malaria Immunology and Vaccinology, Division of Intramural Research, National Institute of Allergy and Infectious Diseases, National Institutes of Health, Twinbrook II Rm 239 A, 12441 Parklawn Drive, Rockville, MD 20852, USA

**Keywords:** Cerebral malaria, Microcirculation, Lactic acid, Sequestration, HRP2, Anaemia, Parasite Biomass

## Abstract

**Objectives:**

To determine whether sequestration of parasitized red blood cells differs between children with uncomplicated and severe *Plasmodium falciparum* malaria.

**Methods:**

We quantified circulating-, total- and sequestered-parasite biomass, using a mathematical model based on plasma concentration of *P. falciparum* histidine rich protein 2, in Gambian children with severe (*n* = 127) and uncomplicated (*n* = 169) malaria.

**Results:**

Circulating- and total-, but not sequestered-, parasite biomass estimates were significantly greater in children with severe malaria than in those with uncomplicated malaria. Sequestered biomass estimates in children with hyperlactataemia or prostration were similar to those in uncomplicated malaria, whereas sequestered biomass was higher in patients with severe anaemia, and showed a trend to higher values in cerebral malaria and fatal cases. Blood lactate concentration correlated with circulating- and total-, but not sequestered parasite biomass. These findings were robust after controlling for age, prior antimalarial treatment and clonality of infection, and over a realistic range of variation in model parameters.

**Conclusion:**

Extensive sequestration is not a uniform requirement for severe paediatric malaria. The pathophysiology of hyperlactataemia and prostration appears to be unrelated to sequestered parasite biomass. Different mechanisms may underlie different severe malaria syndromes, and different therapeutic strategies may be required to improve survival.

## Introduction

Malaria causes around 1 million deaths per year globally.^1^ Clinical features identify those at highest risk of death,^2^, ^3^ but even with appropriate antimalarial therapy, mortality rates remain at least 10–15%, and most deaths occur within 24–48 h of admission.^4^, ^5^ The pathophysiology of severe malaria is poorly understood, and hence the most appropriate supportive care strategies are largely unknown,^6^, ^7^, ^8^, ^9^ and effective adjunctive treatments are lacking.^10^ Better understanding of the pathophysiology of severe malaria might direct better use of simple supportive treatments and reduce the huge burden of death.^11^

Most deaths from malaria occur in African children.^1^ Paediatric severe malaria (SM) comprises several different, sometimes overlapping, syndromes – cerebral malaria (CM), severe anaemia (SA), hyperlactataemia (LA) (or a similar syndrome defined by acidosis or respiratory distress^11^, ^12^) and severe prostration (SP).^13^ CM and LA are common and associated with high risk of death.^2^, ^14^, ^15^, ^16^ The factors that determine why a child develops one rather than another SM syndrome are unknown. Parasitized red blood cells (pRBC) containing mature forms of *Plasmodium falciparum* adhere to vascular endothelium, a phenomenon known as sequestration,^17^ and can cause microvascular obstruction, proposed to be central to the pathogenesis of SM.^11^, ^18^, ^19^ Numerous sequestered pRBCs are found in the cerebral microvasculature of children and adults dying from CM,^20^, ^21^ and correlate with retinal microvascular pathology prior to death.^21^ However, there are no contemporary postmortem studies in severe non-CM syndromes in children, and interpretation of data from postmortem studies is constrained by the absence of control groups with uncomplicated malaria (UM) (who, by definition, survive). Dondorp et al. estimated sequestered-parasite biomass from the plasma concentration of *P. falciparum* histidine rich protein 2 (PfHRP2).^22^ Thai adults with SM had 10-fold higher sequestered-parasite biomass than those with UM,^22^ but the association of sequestration with discrete SM syndromes was not examined. Other observations suggest mechanisms independent of pRBC sequestration may also contribute to SM: *Plasmodium vivax* can cause SM but exhibits little cyto-adherence^23^, ^24^; even in fatal *P. falciparum* CM the degree of sequestration in cerebral vessels and tissues is extremely variable^21^, ^25^; and soluble mediators can also cause endothelial dysfunction and microcirculatory impairment in SM.^11^, ^26^ Surprisingly, pRBC sequestration has never been compared between children with SM and UM controls, despite differences in SM manifestations between children and adults.^13^, ^27^ In the present study we aimed to quantify sequestered-parasite biomass in children with UM and SM.

## Methods

### Recruitment

With approval from the Gambia Government/MRC Laboratories Joint Ethics Committee, and the Ethics Committee of the London School of Hygiene and Tropical Medicine, all samples were collected with informed consent from the child's parent or guardian and used for an unmatched case-control study nested within a larger prospective cohort, of which methodological details have been published.^28^ During each malaria season from August 2007 through January 2011, all Gambian children (<16 years old) presenting to any of three health centres with *P. falciparum* malaria (defined by clinical symptoms and ≥5000 asexual parasites/μL blood) were eligible for recruitment. Clinical management followed Gambian government guidelines, with SM cases admitted to hospital. Blood cultures were not routinely performed, but children were excluded if the attending clinician suspected concomitant bacterial infection. SM was defined using modified WHO criteria^13^: SA, hemoglobin <5 g/dL; LA, blood lactate >5 mmol/L; CM, Blantyre coma score ≤2 for at least 2 h in the absence of hypoglycemia; SP, inability to sit unsupported (children >6 months of age) or inability to suck (children ≤ 6 month). Children fulfilling criteria for both SP and SA, LA, or CM were classified as having SA, LA, or CM rather than SP. Eligible children without signs of SM were classified as UM. On presentation, capillary blood was used to measure lactate and glucose and to prepare thick and thin blood films; venous blood was collected for sickle cell screen, full blood count, and plasma storage (transported to the laboratory on ice within 2 h, separated and stored at −70 °C). Outcome was assessed by survival 7 days after presentation.

### PfHRP2 ELISA and parasite biomass calculation

PfHRP2 was measured in duplicate in plasma by ELISA kit (Cellabs) following the manufacturer's instructions with addition of a standard curve. Laboratory personnel were unaware of the clinical status of subjects. Circulating-, total- (PfHRP2-derived), and sequestered-parasite biomass estimates were calculated using formulas derived by Dondorp et al.^22^ with an initial parasite replication rate of 7.5 (the average estimated in African children with SM),^29^ an elimination constant of 1.26,^30^ and modification of the blood volume term in the equation to improve accuracy for children as follows: males, blood volume (mL) = 312 + (63.11 × body weight (kg)); females, blood volume (mL) = 358 + (62.34 × body weight (kg)).^31^ To account for variation in size of children, parasite biomass was expressed as parasites/kg body weight. Positive and negative values for sequestered biomass occur because the model assumes sampling in mid-erythrocytic cycle such that total biomass may be over- or underestimated in an individual depending on the maturation stage of parasites in their body^22^: in the absence of sequestration, sequestered biomass estimates would form a symmetric distribution around zero.

### Clonality of infection

Clonality of *P. falciparum* infection was assessed as described previously.^32^

### Bacterial co-infection

Bacteraemia with metabolically active *Streptococcus pneumoniae* and non-Typhoid Salmonella (NTS) was determined using quantitative PCR on cDNA.^33^

### Statistical analyses

Statistical analyses were performed using PASW statistics 18 (SPSS Inc.), GraphPad Prism (GraphPad Software Inc.) and the R-statistical software (R Foundation). Data was log_10_ transformed for parametric analyses to achieve normality, except sequestered biomass (comprising positive and negative values) which was analyzed with non-parametric methods. Unpaired *t*-tests and likelihood-ratio tests were used to compare means and medians, respectively, of groups. Confounding by age, prior antimalarial treatment and clonality of infection was assessed by quantile regression (“quantreg” package, R-statistical software): a model including only intercept was compared by means of likelihood-ratio tests to models with any combination of the above covariates or interaction terms. Correlation was assessed using Spearman's rank correlation coefficient. To allow for the multiplicity of tests resulting from multiple responses and multiple comparisons within a response, a false discovery rate (FDR) of 5% was assumed, using the Benjamini and Hochberg approach.^34^ Power of likelihood-ratio tests for detecting an X-fold difference in medians was determined by bootstrapping (10,000 replicates): re-sampled data from the distribution of sequestered-parasite biomass estimates was compared with a similar sample to which (*X* − 1) times the median sequestered biomass was added. Sensitivity analyses assessed the range within which each model parameter could be varied without rejection of the null hypothesis of equal median sequestered biomass among groups. In addition, the effect of joint variation in the parameters was assessed by sampling 10,000 candidate values from uniform distributions within the limits defined for each individual parameter, determining the frequency with which the likelihood-ratio test did not reject the null hypothesis. The effect of parameter variation on the Spearman's rank correlation between lactate and sequestered biomass was assessed identically.

## Results

**Figure 1:**
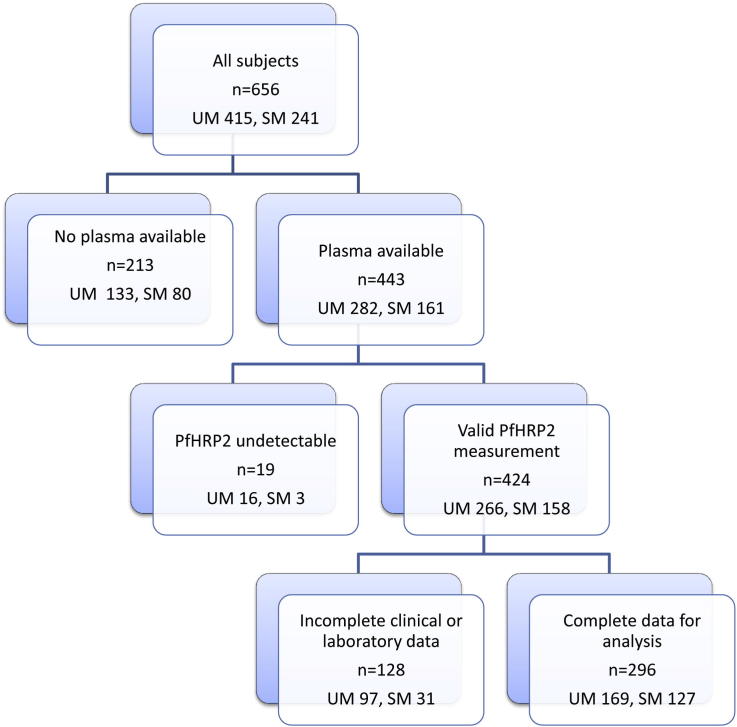
Flow diagram showing selection of subjects included in the study.

**Figure 2:**
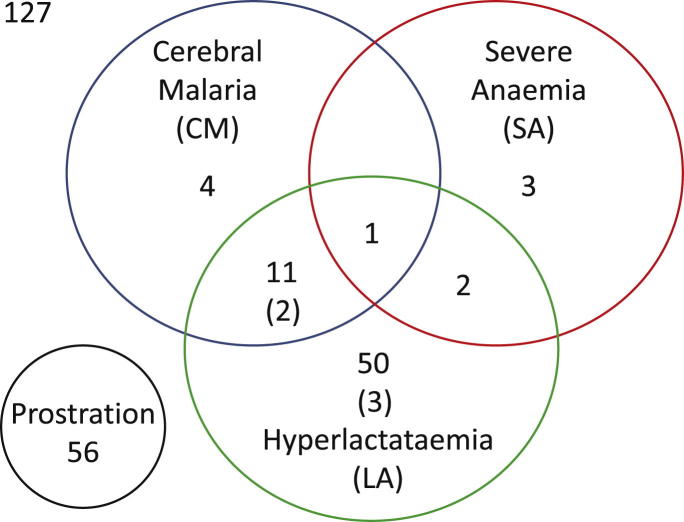
Overlapping manifestations of severe malaria. Number of children (number of deaths, if any, in parentheses) with each severe malaria syndrome. Syndromes were classified inclusively (CM, cerebral malaria; LA, hyperlactataemia; SA, severe anaemia) or exclusively (CM, LA, SA; CMLA, cerebral malaria plus hyperlactataemia; CMSA, cerebral malaria plus severe anaemia; LASA, hyperlactataemia plus severe anaemia; CMLASA, cerebral malaria plus hyperlactataemia plus severe anaemia).

Complete clinical and laboratory data (Fig. 1) were available from 296 children (Tables 1 and 2), 127 (42.9%) with SM, of whom 5 died (Fig. 2).

**Figure 3:**
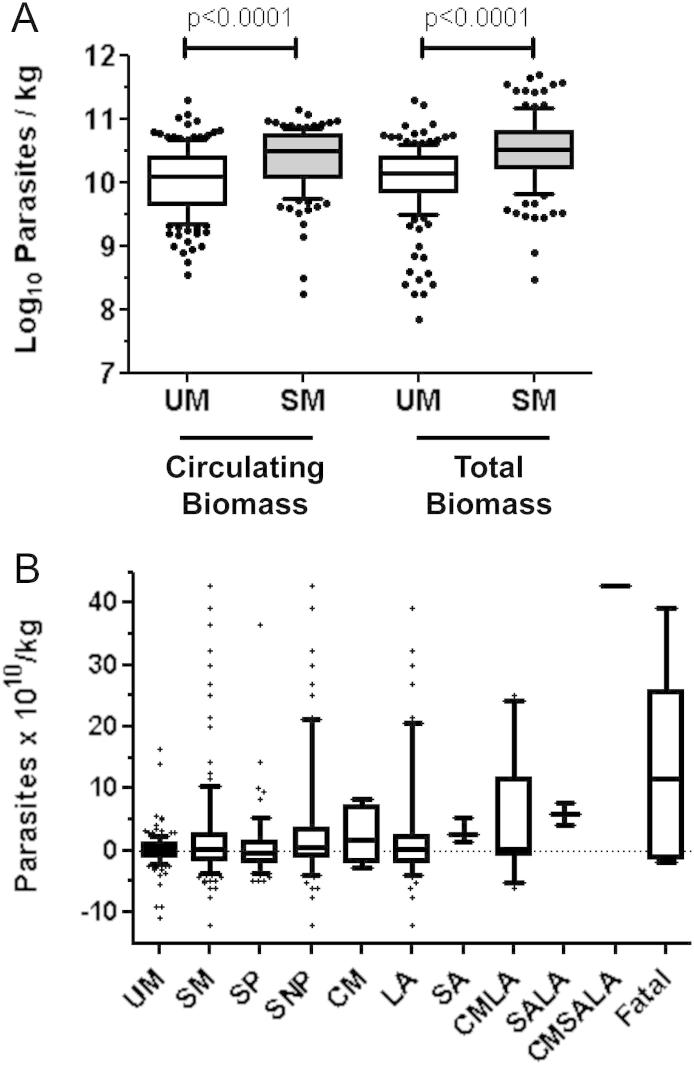
Parasite biomass estimates in uncomplicated and severe malaria. Median (horizontal line), interquartile range (box), 10th and 90th centiles (whiskers). (A) Circulating and total parasite biomass estimates in uncomplicated (*n* = 169) and severe malaria (*n* = 127). (B) Sequestered biomass estimates for uncomplicated (UM) and different manifestations of severe malaria (SM; SP, severe prostrated; SNP, severe non-prostrated; CM, cerebral malaria; LA, hyperlactataemia; SA, severe anaemia; CMLA, cerebral malaria plus hyperlactataemia; CMSA, cerebral malaria plus severe anaemia; LASA, hyperlactataemia plus severe anaemia; CMLASA, cerebral malaria plus hyperlactataemia plus severe anaemia).

Children with SM were younger, more anaemic and thrombocytopaenic, and had higher blood lactate, parasitaemia, parasite density, plasma PfHRP2 concentrations, circulating parasite biomass, and total parasite biomass (calculated from PfHRP2 concentration) than children with UM (Table 2 and Fig. 3A). Sequestered-parasite biomass estimates for individual subjects included both positive and negative values (Fig. 3B), which are possible because the model for total parasite biomass assumes that blood is sampled at a random time point of the erythrocytic cycle of parasite development,^22^ but in reality subjects might present at any time from just after schizogony (when peripheral parasitaemia will be highest) to just before schizogony when peripheral parasitaemia will be lowest. However, comparison of median sequestered-parasite biomass estimates between groups is less affected by the imprecision of estimates for individuals, and sequestered biomass estimates were not significantly different between children with UM and SM (Table 2 and Fig. 3B). This surprising finding prompted us to explore sequestered biomass in subgroups of patients with SM. A large proportion (56 of 127, 44.1%) of SM cases had SP alone, which is associated with a lower risk of in-hospital mortality than the other indicators of severity,^2^ so we reanalyzed the data for subjects with SM excluding those with SP. The median sequestered-parasite biomass in the 71 children with the most severe manifestations of malaria (i.e. CM, SA, LA or any combination of these, collectively termed severe non-prostrated (SNP)) remained not significantly different to that of the UM cases (Table 2, Fig. 3B). To explore whether sequestered biomass was associated with any of the overlapping manifestations of SM, we compared the median sequestered-parasite biomass in children with UM with that in children with each inclusively defined SM syndrome (Table 2). Sequestered biomass in children with LA, the largest subgroup (*n* = 64), was not significantly different to those with UM. In contrast children with CM, SA, and non-survivors, had very high-sequestered biomass, but these subgroups were relatively small, and only in SA cases was the median sequestered biomass significantly higher than that in UM (Table 2, Fig. 3B). We also compared sequestered biomass in exclusively defined SM manifestations with that in children with UM, but the small sizes of the subgroups only allowed us to confirm that median sequestered biomass was similar in UM and LA (Table 2, Fig. 3B). Reanalysis using a less stringent definition of LA (>4 mmol/L, as used by Dondorp et al.^22^), increased the numbers of children classified as having SM, SNP, and LA to 142, 103 and 100 respectively, but did not change the significance of the comparisons between the different categories (Table 3).

Blood lactate concentration is strongly associated with mortality in *P. falciparum* malaria,^14^, ^15^, ^22^ but did not significantly correlate with sequestered-parasite biomass (Spearman *r* = 0.0315, *P* = 0.59), whereas lactate correlated equally well with circulating and total parasite biomass estimates (Spearman *r* = 0.50 (95% CI 0.40–0.58) and 0.44 (95% CI 0.34–0.53) respectively, both *P* < 0.001).

There was no confounding effect of age, prior antimalarial treatment or clonality of infection in the complete data (*P* = 0.95, *P* = 0.11 and *P* = 0.57 respectively): overall, the basic model explained the data best. Misclassification bias, due to bacterial sepsis causing severe disease manifestations in children with co-incidental parasitaemia, was assessed by PCR to detect NTS or *S. pneumoniae* bacteraemia in 160 (54.1%) study subjects with suitable samples (85 of 169 (50.3%) UM and 75 of 127 (59.1%) SM cases); none (95% CI 0–2.3%) were positive. Additionally no study subjects received intravenous antibiotics, making significant misclassification unlikely. Sensitivity analysis revealed that the model was highly robust to a realistic range of variation in parameters (Table 4). The power to detect a 7-fold difference in median sequestered biomass between subjects with UM and SM, UM and SNP, and UM and LA, was 87%, 76%, and 72% respectively.

## Discussion

Improved understanding of the pathophysiology of SM may allow a rational approach to improving supportive care, and provide explanations for why so many interventions to date have proved ineffective or even harmful.^9^, ^10^ Unraveling the pathophysiology of SM is difficult because studies in humans can only describe associations, and cannot prove causality, whilst the relevance of animal models of SM in humans is contentious.^35^ Microcirculatory impairment is often thought to be central to the pathogenesis of both CM and LA,^11^, ^18^, ^19^ but it is not conclusively established whether extensive pRBC sequestration is the primary cause of microcirculatory impairment,^36^ or a consequence of inflammatory endothelial activation and dysfunction which then facilitates pRBC sludging and adherence.^11^, ^17^, ^37^ Furthermore, it is uncertain whether different mechanisms underlie different SM syndromes. The assumption that a single mechanism, pRBC sequestration, causes all SM, led the authors of a recent study to conclude that a “U-shaped” relationship between plasma PfHRP2 and mortality was due to many children with low PfHRP2 concentrations dying from non-malarial causes.^30^ Another explanation for this interesting observation is that SM arises from heterogeneous mechanisms, some related to high parasite burden, and some occurring at lower total parasite burden.

The present study was undertaken to assess the role of one of the most fundamental processes in *P. falciparum* malaria, the sequestration of pRBCs, in causing severe disease. If SM is caused by extensive sequestration of pRBCs within the microvasculature, then the sequestered biomass would be expected to be higher in SM than in UM. We found that all indices of parasite biomass (parasitaemia, parasite density, PfHRP2 concentration, circulating parasite biomass, and total parasite biomass) except the sequestered biomass were higher in SM compared with UM, suggesting that extensive pRBC sequestration does not uniformly underlie SM. This unexpected finding may be explained by the fact that the most frequent SM syndromes in this study were LA and SP: children with these syndromes had very similar median sequestered biomass to those with UM and blood lactate concentrations correlated significantly with circulating biomass (and total biomass) but not sequestered biomass. On the other hand, children with CM, SA and fatal cases had high sequestered biomass; although the small numbers of subjects in these groups in this study preclude firm conclusions, our data are consistent with a role for parasite sequestration in these syndromes. However, the low sequestered biomass in children with LA, and the lack of a significant correlation between sequestered biomass and blood lactate concentrations, is a particularly important observation because LA (or a large base deficit) is one of the most frequent entities defining SM and risk of death in African children with malaria.^2^, ^14^, ^15^, ^16^

Our findings – that overall sequestered-parasite biomass differed only 1.2-fold between SM and UM patients – contrast with findings of an average 10-fold difference between Thai adults with SM and UM in a similarly large study.^22^ The proportions of SM cases with lactate levels above 5 mmol/L were similar in the two studies (44% in Thai adults and 50% in Gambian children), but the proportions with CM differed greatly (64% and 13%, respectively), and this difference may partially explain the discrepant findings. Indeed, in our study the median sequestered biomass in CM cases was 17-fold greater than in the UM cases. In Thai adults, blood lactate concentration correlated significantly with sequestered-parasite biomass but not circulating biomass,^22^ but this relationship may be confounded by the large proportion of cases with CM; unfortunately the data provided in the Thai study does not allow subgroup analysis by discrete SM syndromes.

In assessing the validity of our findings we must consider methodological issues which might influence our results. Plasma concentrations of PfHRP2 in our study were lower than those reported in several other studies of SM,^30^, ^38^, ^39^, ^40^ but the transmission setting appears to influence the plasma PfHRP2 concentrations associated with SM,^30^ and so absolute values should be compared with some caution. In addition the proportions of children with CM, LA and SA were very different to our study, with 29–57% of SM cases in some of these studies having SA, compared with less than 5% of SM cases in our population, and SA was strongly associated with higher levels of PfHRP2.^30^, ^40^ Our study was conducted in children, whereas the model relating PfHRP2 concentration to parasite biomass was derived from data in adults in a different transmission setting.^22^ To account for differences between children and adults we modified relevant model parameters, using data from studies in African children,^29^, ^30^ and we report parasite biomass data relative to body weight. Based on the average replication rate estimated *in vivo* in African children with SM,^29^ we used an initial value for parasite replication rate of 7.5, whereas in Thai adults Dondorp et al. used a value of 8,^22^ and in African children Hendriksen et al. used a value of 3 (based on *in vitro* and non-human primate data).^30^ Importantly, our main findings were robust to variation of this parameter over most of the range of replication rates estimated to occur in African children with SM (as shown in Table 4).^29^ Furthermore, sequestered-parasite biomass in children with CM in our study was quantitatively similar to that estimated mathematically from parasite clearance curves in Gambian children with CM.^41^ In a sensitivity analysis we found that our main conclusions were robust to reasonable variations in model parameters. However, we have made the same assumptions as Dondorp et al., that model parameters are the same for UM and SM, and do not vary during the course of infection.^22^ Although data from humans to inform between-group and temporal variations in model parameters is lacking, recent data from *Plasmodium berghei* ANKA infection in mice suggests that the sequestration rate and the clearance rate of sequestered-parasites may be dynamic throughout the course of an infection,^42^ making this an important area for future study.

It is also important to consider potential sources of bias in our study which might influence our results. Antibodies against PfHRP2 might cause under-estimation of sequestered biomass in SM relative to UM cases, but young Gambian children (who are most likely to have SM) are the least likely to have antibodies to *P. falciparum* antigens.^43^ Prior antimalarial treatment and polyclonal infections might cause deviation from the basic assumptions of the model,^22^ but we found no evidence of any interaction between age, prior antimalarial treatment, or clonality of infection, with severity and sequestered-parasite burden. Differences in parasite multiplication rate between UM and SM cases might be a source of bias.^22^ However, parasite multiplication rate is thought to be reduced at high parasite densities,^44^ which we observed predominantly in SM cases; using the same multiplication rate for UM and SM is thus expected to lead to over- rather than under-estimation of the total parasite biomass in children with SM. Other causes of illness may mimic the clinical features of SM in children with incidental parasitaemia and lead to misclassification. One postmortem study showed that 23% of clinically defined fatal cases of CM had an alternative cause of death,^21^ but there are no comparable data for other SM syndromes or for survivors of SM. By comparison our study was conducted in a relatively low transmission setting^43^ (which reduces the likelihood of incidental parasitaemia),^45^ used a relatively high cut-off peripheral parasitaemia for inclusion (which improves the specificity of diagnosis of malaria),^45^ and we found no evidence of bacterial co-infection, the most likely alternative or confounding cause of severe illness, using specific PCR for the two most common bacterial pathogens.^33^ Two recent studies have reported rates of bacteraemia in children with SM of around 5%^40^, ^46^: one reported lower rates of bacteraemia when parasitaemia thresholds were applied to increase the specificity of the diagnosis of SM; the other did not use a parasitaemia threshold for inclusion, had a high proportion of children with SA (an established risk factor for bacteraemia),^47^, ^48^ and found the risk of bacteraemia was highest in children with very low or very high PfHRP2 concentrations.^40^ We did not assess the presence of malarial retinopathy, which increases the specificity of the diagnosis of CM,^21^ however CM subjects were a relatively small subgroup and amongst those with highest sequestered biomass estimates. Finally, the mortality rate in our study was only 3.9% in SM cases, which might indicate that the children were ‘less’ seriously ill than our SM definitions suggest, but is also consistent with the lower risk of mortality in children,^27^ the proportions of different SM syndromes in our study,^2^ exclusion of children suspected to have non-malarial illness,^28^ and with our subjects living relatively close to the health-care facilities.^28^, ^49^ After considering methodological issues and these sources of bias we believe our findings are robust.

How should our results be interpreted? Although the number of children with SA was small, the association with high PfHRP2 concentration is consistent with other studies,^30^, ^40^ and extensive sequestration could be a causative factor in SA. This would not necessarily require sequestration in the microvasculature, since retention of parasites in the slow open circulation of the spleen would also remove pRBCs from the systemic circulation,^50^ and could explain this observation. Furthermore, we speculate that the role of microvascular obstruction by sequestered pRBCs in SM pathophysiology may differ between the SM syndromes of LA, CM, and SA, and possibly between children and adults. Differences in the pathophysiology of LA and CM are consistent with distinct patterns of risk relative to exposure and age,^51^ additive effects on the risk of mortality,^16^ and differences in the associated pRBC adhesion phenotypes.^52^ LA in malaria is thought to be due to microcirculatory impairment and consequent tissue hypoxia.^6^, ^11^ A recent study demonstrated impairment of the ability of the microvasculature to increase tissue oxygen delivery to match demand in severe malaria, and the severity of this impairment correlated strongly with blood lactate.^53^ Different host and parasite factors may pre-dispose to sequestration-independent microcirculatory dysfunction in LA (perhaps mediated by inflammatory cytokines, hypoargininemia and nitric oxide depletion),^11^, ^26^ whereas pRBC sequestration may be more important in CM. Both mechanisms may have synergistic effects when LA and CM co-exist. A recent large observational study in The Gambia showed that children with LA who received a blood transfusion were much less likely to die than those who did not, suggesting that blood transfusion may be one way to improve tissue oxygen delivery in this group.^54^ Surprisingly though, no randomized trials have been performed to assess the benefit of even this simple intervention.^8^ However, microcirculatory impairment is not the only pathophysiological mechanism occurring in SM, and in common with many other infections, an excessive inflammatory response is considered to contribute to severe disease.^55^ Since PfHRP2 is mainly released at schizogony, its concentration parallels the release of pro-inflammatory parasite molecules such as glycosylphosphatidylinositol and hemozoin from within the pRBC,^56^ and PfHRP2 concentration correlates significantly with C-reactive protein in plasma.^57^ The production of inflammatory cytokines such as TNF-α may be directly involved in the pathophysiology of SM,^37^ and the distribution of pRBCs in the spleen, systemic circulation, or sequestered in specific vascular beds, could influence local concentrations of pro-inflammatory cytokines in, e.g. the brain. Thus interpretation of differences in parasite biomass estimates between SM groups must also be considered alongside concomitant differences in the magnitude and localization of inflammatory stimuli which could influence the presentation of SM. Future studies of malaria pathogenesis and adjunctive treatment should carefully evaluate differences between SM syndromes, and consider the possibility that they require different interventions to improve survival.

## Funding

This work was supported by core funding from the Medical Research Council, UK to the malaria research programme, and a Medical Research Council, UK, clinical research training fellowship [G0701427 to A.J.C.].

## Conflict of interest

The authors have no commercial or other association that might pose a conflict of interest.

## Figures and Tables

**Table 1:** Characteristics of children enrolled in the study.

	Uncomplicated malaria		Severe malaria	
*n*	%	*n*	%
All children	169	57.1	127	42.9

Sex				
Male	93	55.0	76	59.8
Female	76	45.0	51	40.2

Ethnicity				
Mandingo	64	37.9	53	41.7
Wollof	19	11.2	9	7.1
Fulla	34	20.1	24	18.9
Jola	31	18.3	19	15.0
Serehuli	1	0.6	1	0.8
Serere	6	3.6	4	3.1
Manjago	7	4.1	6	4.7
Aku	1	0.6	0	0
Other	4	2.4	6	4.7
Unknown	2	1.2	5	3.9

Prior antimalarial				
Yes	24	14.2	29	22.8
No	136	80.5	92	72.4
Unknown	9	5.3	6	4.7

Clonality of infection				
Monoclonal	48	28.4	28	22.0
Polyclonal	67	39.6	49	38.6
Not tested	54	32.0	50	39.4

Survival to day 7				
Yes	169	100	122	96.1
No	0	0	5	3.93

**Table 2:** Comparison of indicators of severity and parasite biomass in uncomplicated and severe malaria.*

Clinical manifestation^a^	*n* (%)	Age (years) GM (95%CI)	Hemoglobin (g/dL) GM (95%CI)	Platelets (×10^9^/L)^b^ GM (95%CI)	Lactate (mmol/L) GM (95%CI)	Parasitaemia (%) GM (95%CI)	Parasite density (×10^5^ /μL) GM (95%CI)	Plasma PfHRP2 (ng/mL) GM (95%CI)	Circulating parasite biomass (×10^10^/kg) GM (95%CI)	Total parasite biomass (×10^10^/kg) GM (95%CI)	Sequestered biomass (×10^10^/kg)^c^ median (95%CI)
Uncomplicated	169 (57.1)	6.47 (5.88–7.12)	11.20 (10.90–11.51)	109.6 (98.7–121.7)	2.10 (1.97–2.24)	3.00 (2.52–3.58)	1.29 (1.08–1.53)	101.5 (84.43–122.1)	1.04 (0.87–1.24)	1.13 (0.93–1.37)	0.099 (−0.10 to 0.37)
All severe	127 (42.9)	4.25 (3.89–4.64) ***P* < 0.001**	9.11 (8.69–9.55) ***P* < 0.001**	61.2 (52.7–71.2) ***P* < 0.001**	4.69 (4.14–5.32) ***P* < 0.001**	7.90 (6.49–9.61) ***P* < 0.001**	2.79 (2.30–3.38) ***P* < 0.001**	247.6 (202.1–303.3) ***P* < 0.001**	2.38 (1.97–2.89) ***P* < 0.001**	3.14 (2.54–3.89) ***P* < 0.001**	0.12 (−0.27 to 0.84) *P* = 0.89

Severe											
SP	56 (18.9)	4.23 (3.71–4.82) ***P* < 0.001**	9.66 (9.09–10.25) ***P* < 0.001**	62.2 (50.8–76.2) ***P* < 0.001**	2.88 (2.37–3.50) ***P* < 0.001**	5.97 (4.34–8.21) ***P* < 0.001**	2.25 (1.64–3.09) ***P* = 0.002**	191.3 (146.6–249.7) ***P* < 0.001**	1.94 (1.41–2.66) ***P* < 0.001**	2.39 (1.80–3.16) ***P* < 0.001**	−0.29 (−0.71 to 0.57) *P* = 0.30
SNP	71 (24.0)	4.27 (3.77–4.83) ***P* < 0.001**	8.70 (8.11–9.34) ***P* < 0.001**	60.5 (48.5–75.6) ***P* < 0.001**	6.90 (6.27–7.60) ***P* < 0.001**	9.85 (7.70–12.6) ***P* < 0.001**	3.31 (2.60–4.20) ***P* < 0.001**	303.4 (225.2–408.8) ***P* < 0.001**	2.81 (2.21–3.58) ***P* < 0.001**	3.90 (2.85–5.33) ***P* < 0.001**	0.57 (−0.04 to 1.44) *P* = 0.20

Inclusive category											
CM	16 (5.4)	3.99 (3.16–5.05) ***P* = 0.003**	8.60 (7.38–10.02) ***P* < 0.001**	39.9 (20.5–77.9) ***P* < 0.001**	7.31 (5.48–9.75) ***P* < 0.001**	11.38 (7.44–17.4) ***P* < 0.001**	3.60 (2.45–5.31) ***P* < 0.001**	417.4 (211.8–822.7) ***P* < 0.001**	3.10 (2.11–4.55) ***P* = 0.001**	5.52 (2.74–11.1) ***P* < 0.001**	1.69 (−0.49 to 8.45) *P* = 0.16
LA	64 (21.6)	4.38 (3.83–5.00) ***P* < 0.001**	8.89 (8.30–9.52) ***P* < 0.001**	58.1 (45.8–73.8) ***P* < 0.001**	7.52 (6.95–8.13) ***P* < 0.001**	10.33 (7.94–13.4) ***P* < 0.001**	3.55 (2.75–4.59) ***P* < 0.001**	312.4 (226.0–431.9) ***P* < 0.001**	3.01 (2.32–3.91) ***P* < 0.001**	3.98 (2.83–5.60) ***P* < 0.001**	0.36 (−0.19 to 1.30) *P* = 0.49
SA	6 (2.0)	2.75 (1.46–5.17) ***P* = 0.001**	4.54 (4.20–4.91) ***P* < 0.001**	79.7 (43.5–146.1) *P* = 0.27	5.31 (2.25–12.49) ***P* < 0.001**	10.17 (5.38–19.2) ***P* = 0.01**	1.87 (0.98–3.59) *P* = 0.43	484.8 (169.6–1386) ***P* = 0.002**	1.69 (0.92–3.12) *P* = 0.31	7.84 (2.82–21.8) ***P* < 0.001**	4.64 (2.08–25.2) ***P* < 0.001**

Exclusive category											
CM	4 (1.4)	3.76 (2.15–6.57) *P* = 0.09	10.06 (7.19–14.09) *P* = 0.24	81.7 (21.9–304.6) *P* = 0.41	3.52 (2.33–5.31) ***P* = 0.02**	4.29 (1.01–18.3) *P* = 0.54	1.62 (0.46–5.69) *P* = 0.69	203.4 (38.0–1118) *P* = 0.26	1.39 (0.41–4.70) *P* = 0.62	2.53 (0.43–15.1) *P* = 0.21	1.69 (−2.66 to 8.48)
LA	50 (16.9)	4.62 (3.99–5.35) ***P* < 0.001**	9.32 (8.72–9.96) ***P* < 0.001**	66.3 (52.6–83.6) ***P* < 0.001**	7.12 (6.56–7.74) ***P* < 0.001**	9.46 (6.83–13.1) ***P* < 0.001**	3.43 (2.50–4.69) ***P* < 0.001**	271.2 (187.2–392.7) ***P* < 0.001**	2.88 (2.09–3.97) ***P* < 0.001**	3.37 (2.28–4.97) ***P* < 0.001**	0.07 (−0.35 to 1.12) *P* = 0.99
SA	3 (1.0)	2.89 (1.41–5.89) ***P* = 0.03**	4.55 (3.61–5.74) ***P* < 0.001**	96.3 (16.7–554.6) *P* = 0.75	2.76 (1.04–7.34) *P* = 0.26	10.73 (4.23–27.2) *P* = 0.06	1.85 (0.54–6.35) *P* = 0.59	277.1 (61.8–1242) *P* = 0.16	1.63 (0.49–5.37) *P* = 0.51	4.38 (1.03–18.6) *P* = 0.05	2.71 (1.44–5.23)
CMLA	11 (3.7)	3.94 (2.84–5.46) ***P* = 0.01**	8.63 (7.37–10.11) ***P* < 0.001**	30.9 (12.3–77.7) ***P* < 0.001**	8.82 (7.21–10.81) ***P* < 0.001**	15.72 (11.7–21.2) ***P* < 0.001**	4.96 (3.59–6.85) ***P* < 0.001**	453.1 (199.8–1027) ***P* < 0.001**	4.28 (3.13–5.87) ***P* < 0.001**	6.06 (2.65–13.8) ***P* < 0.001**	0.33 (−0.55 to 11.5) *P* = 0.48
LASA	2 (0.7)	1.73	4.59	87.0	7.82	7.48	1.62	450.6	1.58	7.85	5.81
CMLASA	1 (0.3)	6.0	4.40	38.0	17.3	16.0	2.62	3005	2.18	45.0	42.9
Non-survivors	5 (1.7)	6.26 (3.40–11.54) *P* = 0.91	9.31 (6.79–12.76) ***P* = 0.03**	26.0 (18.3–36.9) ***P* < 0.001**	9.31 (5.90–14.69) ***P* < 0.001**	14.42 (5.19–40.0) ***P* = 0.003**	5.12 (2.25–11.6) ***P* = 0.009**	763.4 (162.6–3584) ***P* < 0.001**	4.14 (1.82–9.34) ***P* = 0.009**	9.15 (1.66–50.3) ***P* < 0.001**	11.5 (−2.00 to 39.1)

GM, geometric mean; 95% CI, 95% confidence interval

*P value for comparison with the uncomplicated malaria group using the unpaired *t*-test on log_10_-transformed data, when *n* ≥ 3, unless specified otherwise. Only tests with *P* ≤ 0.029 have a false discovery rate of 5% or less using the Benjamini and Hochberg method to control for multiple comparisons and are considered significant (*P* value in bold type).

a Severe malaria cases were grouped as SP, severe prostrated, or SNP, severe non-prostrated (within which syndromes were grouped inclusively (CM, cerebral malaria; LA, hyperlactataemia; SA, severe anaemia) or exclusively (CM, cerebral malaria; LA, hyperlactataemia; SA, severe anaemia; CMLA, cerebral malaria plus hyperlactataemia; CMSA, cerebral malaria plus severe anaemia; LASA, hyperlactataemia plus severe anaemia; CMLASA, cerebral malaria plus hyperlactataemia plus severe anaemia)).

b Platelet count was not available for four subjects with prostration.

c *P* values for comparison with the uncomplicated malaria group using the likelihood-ratio test when *n* > 5.

**Table 3:** Comparison of indicators of severity and parasite biomass in uncomplicated and severe malaria when hyperlactatemia redefined as lactate >4 mmol/L.*

Clinical manifestation^a^	*n* (%)	Age (years) GM (95%CI)	Hemoglobin (g/dL) GM (95%CI)	Platelets (×10^9^/L)^b^ GM (95%CI)	Lactate (mmol/L) GM (95%CI)	Parasitaemia (%) GM (95%CI)	Parasite density (×10^5^ /μL) GM (95%CI)	Plasma PfHRP2 (ng/mL) GM (95%CI)	Circulating parasite biomass (×10^10^/kg) GM (95%CI)	Total parasite biomass (×10^10^/kg) GM (95%CI)	Sequestered biomass (×10^10^/kg)^c^ median (95%CI)
Uncomplicated	154 (52.0)	6.68 (6.06–7.36)	11.34 (11.04–11.66)	110.6 (99.2–123.4)	1.95 (1.84–2.07)	2.88 (2.40–3.46)	1.23 (1.02–1.49)	99.1 (82.0–119.6)	0.995 (0.83–1.20)	1.09 (0.90–1.33)	0.06 (−0.16 to 0.35)
Severe	142 (48.0)	4.29 (3.93–4.69) ***P* < 0.001**	9.19 (8.79–9.60) ***P* < 0.001**	64.5 (56.0–74.4) ***P* < 0.001**	4.66 (4.17–5.21) ***P* < 0.001**	7.46 (6.18–9.01) ***P* < 0.001**	2.69 (2.24–3.22) ***P* < 0.001**	231.4 (189.4–282.8) ***P* < 0.001**	2.29 (2.09–3.14) ***P* < 0.001**	2.92 (2.37–3.60) ***P* < 0.001**	0.22 (−0.14 to 0.79) *P* = 0.71
SNP	103 (34.8)	4.23 (3.81–4.70) ***P* < 0.001**	8.92 (8.46–9.40) ***P* < 0.001**	67.8 (57.0–80.6) ***P* < 0.001**	6.04 (5.59–6.52) ***P* < 0.001**	8.45 (6.84–10.4) ***P* < 0.001**	3.00 (2.45–3.66) ***P* < 0.001**	254.3 (196.9–328.3) ***P* < 0.001**	2.56 (2.09–3.14) ***P* < 0.001**	3.26 (2.49–4.25) ***P* < 0.001**	0.40 (−0.09 to 0.92) *P* = 0.39
LA	100 (33.8)	4.28 (3.84–4.75) ***P* < 0.001**	8.97 (8.52–9.43) ***P* < 0.001**	65.7 (55.1–78.3) ***P* < 0.001**	6.22 (5.79–6.68) ***P* < 0.001**	8.66 (6.99–10.7) ***P* < 0.001**	3.10 (2.53–3.80) ***P* < 0.001**	262.5 (202.0–341.3) ***P* < 0.001**	2.63 (2.15–3.26) ***P* < 0.001**	3.36 (2.55–4.41) ***P* < 0.001**	0.36 (−0.09 to 1.01) *P* = 0.45

GM, geometric mean; 95% CI, 95% confidence interval

*P value for comparison with the uncomplicated malaria group using the unpaired *t*-test on log_10_-transformed data, when *n* ≥ 3, unless specified otherwise. Only tests with *P* ≤ 0.029 have a false discovery rate of 5% or less using the Benjamini and Hochberg method to control for multiple comparisons and are considered significant (*P* value in bold type).

a Severe malaria cases were considered together, or in subgroups: SNP, severe non-prostrated, and LA, hyperlactataemia.

b Platelet count was not available for four subjects with prostration.

c *P* values for comparison with the uncomplicated malaria group using the likelihood-ratio test when *n* > 5.

**Table 4:** Sensitivity analysis for variation in model parameters.

	Range of variation^a^			Robustness to joint variation^b^
Multiplication rate, *M* (/asexual cycle)	Elimination constant, *k* (/asexual cycle)	Rate of PfHRP2 secretion, *R* (×10^−15^ pg/cycle)
Initial model value	7.5	1.26	5.2	
UM vs. SM	51–149% (3.8–11.2)	0–170% (0–2.14)	75–180% (3.9–9.36)	64%
UM vs. SNP	25–116% (1.88–8.7)	0–126% (0–1.59)	89–365% (4.63–18.98)	47%
UM vs. all LA	35–136% (2.6–10.2)	0–153% (0–1.93)	80–256% (4.16–13.31)	60%
Correlation between lactate and sequestered biomass^c^	58–137% (4.34–10.3) − *r* +	18–155% (0.23–1.95) − *r* +	80–158% (3.71–8.22) + *r* −	63%

UM, uncomplicated malaria; SM, severe malaria; SNP, severe non-prostrated; LA, hyperlactataemia.

a Range of variation (absolute values) within which a likelihood-ratio test does not reject the null hypothesis that the median sequestered-parasite biomass in the comparison groups is equal.

b Robustness (% of simulations in which the null hypothesis is not rejected) to joint variation of the parameters within their individually determined ranges of variation.

c Using Spearmann's rank correlation test, the sign of the correlation coefficient, *r*, is presented for the limits of the parameter range.
